# Synthesis of Polyphosphate Flame Retardant Bisphenol AP Bis(Diphenyl Phosphate) and Its Application in Polycarbonate/Acrylonitrile-Butadiene-Styrene

**DOI:** 10.3390/ma17235682

**Published:** 2024-11-21

**Authors:** Yang Yang, Chunzhi Wang, Yong Guan, Dafu Wei, Xiang Xu

**Affiliations:** Shanghai Key Laboratory of Advanced Polymeric Materials, School of Materials Science and Engineering, East China University of Science and Technology, Shanghai 200237, China

**Keywords:** polycarbonate, acrylonitrile-butadiene-styrene, flame retardant, heat resistance

## Abstract

The flame retardant bisphenol AP bis(diphenyl phosphate) (BAPDP) is synthesized from triphenyl phosphate and bisphenol AP via transesterification, producing a polycarbonate/acrylonitrile-butadiene-styrene (PC/ABS) with a high flame retardancy and thermal stability. In this study, flame-retardant PC/ABS blends with various BAPDP contents are prepared, and their flame retardancy is studied using the limit oxygen index, vertical combustion, thermogravimetric analysis, and cone calorimeter testing. With a BAPDP content of 20 wt%, the product exhibits a limiting oxygen index of 25.4% and achieves the UL-94 V-0 grade, with a thermal deformation temperature of 72.6 °C. BAPDP improves the flame retardancy of the PC/ABS blends and exhibits fewer adverse effects on the thermal deformation temperature than other commercial flame retardants at the same concentration.

## 1. Introduction

Polycarbonate/acrylonitrile-butadiene-styrene (PC/ABS) blends have numerous technical applications owing to the complementary properties of the components and their low costs [1,2]. PC exhibits excellent gloss, along with high impact resistance, heat resistance, transparency, and dimensional stability; however, it suffers from certain disadvantages such as notch sensitivity. Similarly, ABS exhibits high impact resistance and processing fluidity; however, its application is limited owing to its relatively poor heat resistance and mechanical properties. PC and ABS have complementary properties; thus, PC/ABS composites prepared via the blending and extrusion of PC and ABS typically exhibit high chemical resistance, impact resistance, and fluidity. Therefore, PC/ABS has been widely used in the electrical and electronic industries [3]. However, it is highly flammable in air. To expand its use in automotive parts, 5G communication equipment, and office equipment, the flame-retardant properties of PC/ABS must be enhanced. In past decades, adding halogenated flame retardants to PC/ABS was a common modification method [4]. Halogenated flame retardants are typically mixed with Sb_2_O_3_, the combustion of which produces gaseous antimony halides that inhibit oxygen access. Similarly, antimony oxyhalide intermediates produced during the formation of antimony halides interfere with free radical reactions during combustion [5]. While halogenated flame retardants are highly effective, the combustion process produces poisonous gases that are harmful to human health and the environment [6]. Moreover, exposure to halogenated flame retardants may cause several diseases associated with endocrine disruption [7]. Under the ‘Waste Electrical and Electronic Equipment’ and ‘Restriction of Hazardous Substances’ directives, bromine, antimony, and other commonly used halogen flame retardants are banned in the European Union. Accordingly, halogenated flame retardants are being replaced by halogen-free flame retardants, which are more environmentally sustainable, less toxic, and highly efficient [8,9,10].

Halogen-free flame retardants have recently attracted considerable attention owing to their environmental sustainability [11,12,13]. Phosphazene compounds typically exhibit a lower toxicity. For example, hexaphenoxycyclotriphosphazene (HPCTP) presents flame-retardant properties through condensed-phase and gas-phase mechanisms. During combustion, HPCTP decomposes into phosphoric acid, polyphosphoric acid, and N_2_ non-flammable gases. This promotes the dehydration of PC/ABS into a carbonaceous char that expands to form an intumescent char layer, thereby inhibiting heat transfer and oxygen access [14]. However, HPCTP exhibits a relatively low flame-retardant efficiency, requiring the addition of 30 wt% to achieve the UL-94 V-0 grade [15,16]. Meanwhile, silicon is an ecofriendly flame retardant. Zong et al. [17] prepared montmorillonite (MMT) via intercalation and added 5 wt% MMT to PC/ABS (57:38), providing an excellent thermal insulator and mass transfer barrier. The nano-dispersed silicate layer slowed the decomposition rate, increased the degradation temperature, increased the thermal stability, and decreased the flammability. Wang et al. [18] used polymethylphenylsilsesquioxane (PMPSQ) as flame-retardant polymer. The results indicated that PMPSQ enhanced the thermal stability and flame retardancy of the polymer by increasing the activation energy of the system. Zhang et al. [19] studied the flame-retardant mechanism of a novel polyhedral oligomeric silsesquioxane containing a 9,10-dihydro-9-oxa-10-phosphaphenanthrene-10-oxide (DOPO-POSS) compound in PC/ABS alloys. They found that 10 wt% DOPO-POSS enhanced the flame retardancy and thermal stability of the alloys. However, silicon flame retardants have poor flame retardancy and low compatibility with polymer materials. Nano-flame retardants can not only improve a polymer’s flame-retardant rating but also maintain or improve its physical and mechanical properties. Pawlowski et al. [20] added nano-dispersed boehmite (AlOOH) and aryl phosphates to a flame-retardant PC/ABS compound. The results indicated that the flame was restrained, and the limiting oxygen index (LOI) was improved. Pour et al. [21] studied the flame-retardant effect of graphene on PC/ABS alloy. The results suggested that the addition of graphene significantly improved the thermal stability and mechanical properties of the alloy; graphene formed a dense carbon layer in the condensed phase, which improved the flame retardancy. However, the mode of action of the nano-flame retardants has not been explained systematically—particularly the effects of the type and dosage of the flame retardants and the matrix [22,23,24].

PC/ABS alloys are typically mixed with organophosphorus flame retardants [25]. Phosphorus-containing flame retardants exhibit excellent flame retardancy while producing no toxic gases. Therefore, they are among the most promising environmentally sustainable flame retardants [26]. At high temperatures, phosphorus-based flame retardants form a protective layer that covers the lower polymer matrix, thereby achieving excellent flame retardancy. Several studies have indicated the utility of polyphosphate as a flame retardant [27]. Phosphorus-containing flame retardants significantly reduce the flammability of polymer materials and increase the char yield from combustion by altering the decomposition reaction and by forming a protective surface layer of carbon during a fire before the unburned structural material begins to decompose [28,29,30,31]. Aromatic phosphates, such as resorcinol bis(diphenyl phosphate) (RDP), significantly improve the flame retardancy of PC/ABS alloys. In commercial applications, RDP is homogenously mixed with the PC phase, resulting in PC/ABS composites with superior mechanical properties. However, RDP significantly affects the thermal deformation temperature of PC/ABS. For example, RDP can easily absorb moisture, leading to the aging degradation of PC/ABS. Levchik et al. [32] added 9.3 wt% to PC/ABS (5:1), and the thermal deformation temperature decreased from 110.0 to 82.0 °C. Levchik et al. [33] added 14.0 wt% to PC/ABS (3:1), and the thermal deformation temperature decreased to 76 °C. Therefore, the addition of RDP has a significant effect on the thermal deformation temperature of PC/ABS, and balancing flame retardancy and heat resistance is challenging.

In this study, phosphorus flame-retardant bisphenol AP bis(diphenyl phosphate) (BAPDP) is synthesized by the catalytic transesterification of triphenyl phosphate and bisphenol AP. The flame retardancy and the thermal and mechanical properties of PC/ABS are studied. BAPDP contains more benzene rings than other flame retardants, such as the commercially available RDP, which may increase the rigidity of the molecule and improve its heat resistance and flame retardancy. Therefore, the flame retardant BAPDP is expected to improve the flame retardancy of the PC/ABS blends while having fewer adverse effects on the thermal deformation temperature of PC/ABS.

## 2. Materials and Methods

### 2.1. Materials

Bisphenol AP (SPAP) was purchased from Jiangsu Runfeng Synthetic Technology Co., Ltd., Suzhou, China. Triphenyl phosphate (TPP) was acquired from Suzhou New Material Technology Co., Ltd., Suzhou, China. RDP was purchased from Shouguang Derun Chemistry Co., Ltd., Shouguang, China. PC was purchased from Shanghai Qichen Plasticizing Co., Ltd., Shanghai, China. ABS was acquired from Shanghai Junlin Plastics Co., Ltd., Shanghai, China. Sodium phenolate was purchased from Shanghai Myrell Biochemical Technology Co., Ltd., Shanghai, China. Polytetrafluoroethylene (PTFE) was acquired from Jiangsu Anhuai Chemical Technology Co., Ltd., Nantong, China.

### 2.2. Synthesis of BAPDP

**Scheme 1:**
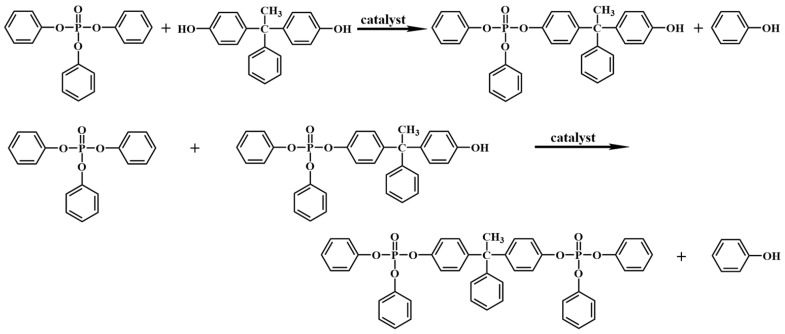
Synthesis of BAPDP.

The synthesis of BAPDP is shown in Scheme 1. TPP (23.4 g, 71.8 mmol) was heated to approximately 70 °C to obtain a colorless solution before SPAP (10.2 g, 35.2 mmol) was added, and the mixture was stirred for approximately 1 h. Sodium phenolate (0.2 g, 1.7 mmol) was then added, and the mixture was stirred for 30 min before the temperature was gradually increased to 220–230 °C over 1.5 h to evaporate the phenol. Lastly, the desired product (21.6 g) was obtained with an 81.5% yield. The product may have contained trace amounts of residual triphenyl phosphate and sodium phenolate (less than 1 wt% of the feed amount) and was used without purification.

### 2.3. Preparation of PC/ABS/BAPDP Composites

PC, ABS, and the anti-dropping agent PTFE were vacuum-dried at 100 °C for 12 h before the raw materials (PC, ABS, BAPDP, and PTFE) were blended in a mixer (Su70-1, Changzhou Suyan Technology Co., Ltd., Changzhou, China) at 210 °C and 60 rpm for 15 min. The compositions of the PC/ABS/BAPDP composites are listed in Table 1. The samples were molded in a plate vulcanizer (XQLB-350 × 350 Shanghai Rubber Machinery, Shanghai, China) at 210 °C for 10 min. The pressed material sheets were cut into splines for the experiments.

### 2.4. Characterization

Fourier-transform infrared (FT-IR) spectroscopy. The samples were prepared using the KBr tablet pressing method, and the spectra were recorded at a resolution of 2 cm^−1^ between 4000 and 400 cm^−1^, using an infrared spectrometer (Nicolet 5700, Nicolet, Green Bay, WI, USA).

The proton nuclear magnetic resonance (^1^H-NMR) spectrum was obtained using a 400 MHz NMR Spectrometer (Ascend 400 NMR, Bruker, Fällanden, Switzerland) using CDCl_3_ as the solvent.

Element analysis. The elemental composition of the material was detected using an elemental analyzer (Vario el Cube, Langenselbold, Germany) and X-ray photoelectron spectroscopy analysis (XPS, ESCALAB 250XI, Thermo Fisher Ltd., Waltham, MA, USA).

Thermogravimetric analysis (TGA). TGA was performed using a comprehensive thermal analyzer (STA 409PC/PG, NETZSCH, Selb, Germany). For each test, approximately 5 mg of the sample was placed in a crucible. The temperature range was set at 25 °C to 600 °C, and the heating rate was 10 °C/min. The tests were performed under a nitrogen atmosphere.

Scanning electron microscopy (SEM). The distribution of the samples was observed using SEM (S-4800, Hitachi, Ltd., Tokyo, Japan) at an accelerating voltage of 10 kV.

The LOI was measured using an oxygen index meter (F101D, Shanghai Qianshi Precision Electromechanical Technology Co., Ltd., Shanghai, China). Each sample had dimensions of 130 × 6.5 × 3 mm^3^, and the results are presented as the average of five measurements.

Vertical combustion test (UL-94). The UL-94 test was performed using a combustion instrument (CZF-2, Nanjing Jiangning Analysis Instrument Co., Ltd., Nanjing, China) in accordance with the ASTMD3801 standard [34]. The samples measuring 125 × 12.5 × 3 mm^3^ were cut, and the results are presented as the average of five measurements.

Cone calorimeter testing (CCT). CCT was performed at a heat flux of 35 kW/m^2^ using a cone calorimeter (Stanton Redcroft, Polymer Laboratories Co., Ltd., London, UK), in accordance with ASTM E 1354-94. The splines were formed into samples measuring 100 × 100 × 4 mm^3^, and the results are presented as the average of three measurements.

The tensile strength of all the samples was measured at a tensile speed of 5 mm·min^−1^. The bending strength was measured at a tensile speed of 2 mm·min^−1^, with a span of 64 mm and a sample size of 80 × 10 × 4 mm^3^. The notch impact strength was determined using samples measuring 80 × 10 × 4 mm^3^ with a 2 mm notch depth. All the tests were repeated five times to obtain an average value.

## 3. Results and Discussion

### 3.1. Characterizations of BAPDP

**Figure 1:**
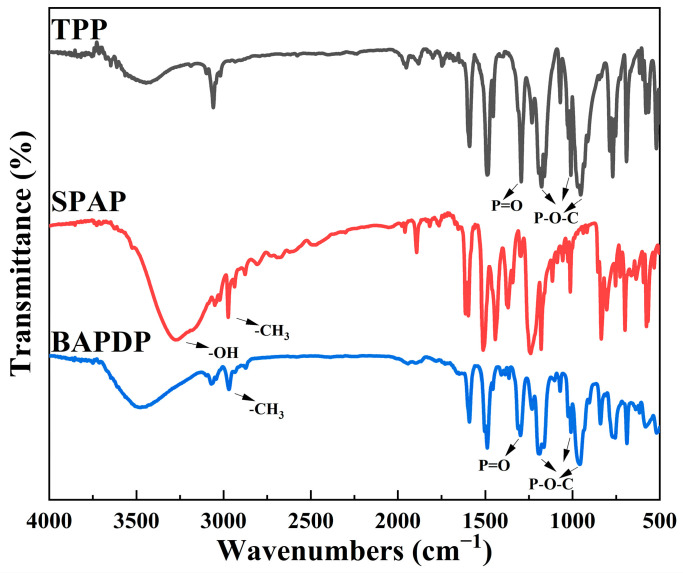
FT-IR spectra of triphenyl phosphate, bisphenol AP, and BAPDP.

The FT-IR spectrum of BAPDP (Figure 1) depicts an absorption band at 3070 cm^−1^, which is attributed to the C-H stretching vibration of the benzene ring of BAPDP. The additional bands at 2969, 1589, and 1489 cm^−1^ are attributed to the methyl C-H stretching vibrations and C-C stretching vibrations in the benzene ring skeleton. The band at 1298 cm^−1^ is attributed to the P=O stretching vibration, and those at 1188, 1009, and 958 cm^−1^ are attributed to the vibrations of P-O-C. The characteristic -OH absorption peak was not observed in the spectrum of BAPDP. The characteristic absorption peaks in the FT-IR spectra of TPP, SPAP, and BAPDP indicated the successful synthesis of BAPDP.

**Figure 2:**
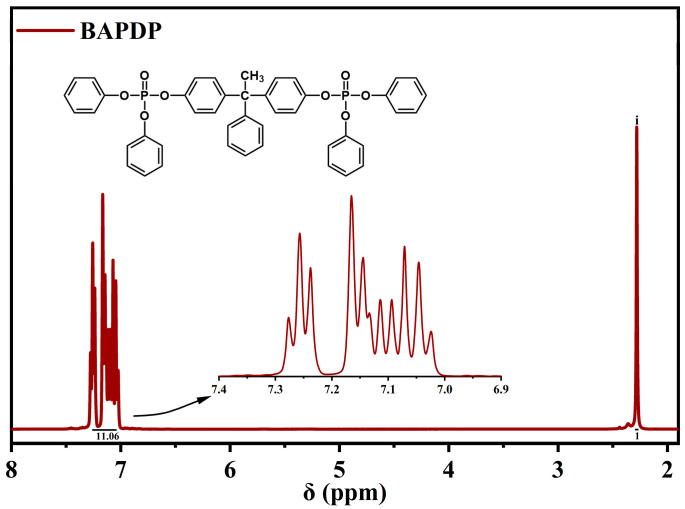
^1^H-NMR spectrum of BAPDP.

The ^1^H-NMR spectrum of BAPDP is depicted in Figure 2. The peaks at chemical shifts of 2.28 ppm and within 7.02–7.28 ppm are indicative of methylene group protons and those of the benzene ring in BAPDP. Moreover, the ratio of the integral of the peaks was approximately 1:11, which was consistent with the theoretical ratio. Therefore, the ^1^H-NMR spectrum indicated the successful synthesis of BAPDP.

The purity of the resulting BAPDP was evaluated through elemental analysis. The elemental contents of C, H, and P concurred well with the theoretical values (Table 2). The slight difference can be attributed to the slight excess of the residual sodium phenolate and the triphenyl phosphate added in slight excess. These trace impurities do not significantly affect the flame retardancy; thus, BAPDP was used without further purification.

**Figure 3:**
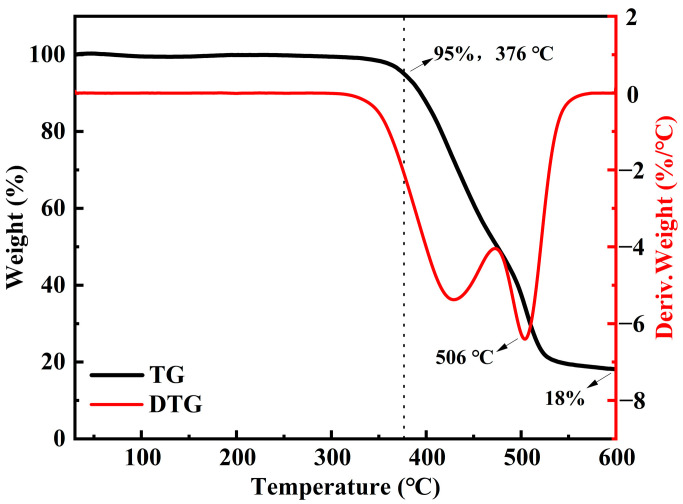
TGA and DTG curves of BAPDP.

Figure 3 shows the TGA and DTG curves of BAPDP. BAPDP experienced a weight loss of 5% at 376 °C, with a peak DTG temperature of 506 °C, demonstrating its excellent high-temperature stability. The TGA curve demonstrated that the BAPDP mass decreased as the temperature increased, with a residual char content of 18% at 600 °C. This indicates that BAPDP is sufficiently stable for application as a flame retardant in PC/ABS composites.

### 3.2. Effect of BAPDP on Flame Retardancy of PC/ABS

**Figure 4:**
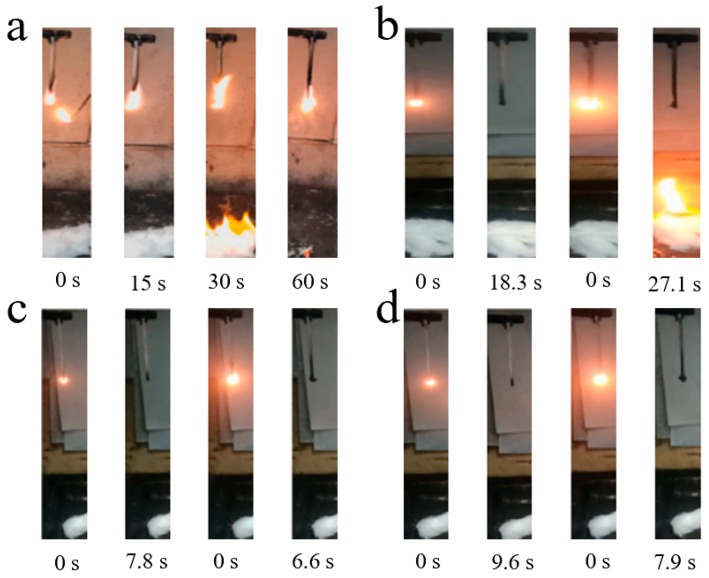
UL-94 vertical burning photographs: (**a**) PC/ABS, (**b**) PC/ABS/10BAPDP, (**c**) PC/ABS/25BAPDP, and (**d**) PC/ABS/25RDP.

The LOI and UL-94 analyses of the flame-retardant PC/ABS are presented in Table 3. PC/ABS presented a low LOI value of 21.1%, along with noticeable melt dripping that could ignite cotton during combustion (Figure 4a). The PC/ABS/15BAPDP and PC/ABS/15RDP exhibited LOI values of 24.7% and 24.1%, respectively, passing the UL-94 V-1 and V-2 grades. In the burning tests for materials with BAPDP and RDP contents of >15 and >20 wt%, respectively, no dripping was observed during combustion. In addition, less time was required for PC/ABS/BAPDP to extinguish the flame than for PC/ABS/RDP in the UL-94 test (Figure 4c,d). Thus, BAPDP has a superior flame-retardant effect corresponding to RDP. The flame retardancy of the samples significantly increased with the BAPDP content; >20 wt%, the LOI of the products was >25% and exceeded the UL-94 V-0 grade. This was attributed to the phosphorous groups of BAPDP, which promote the formation of a dense carbon layer on the PC/ABS surface that further insulates heat and oxygen, thereby achieving a flame-retardant effect.

**Figure 5:**
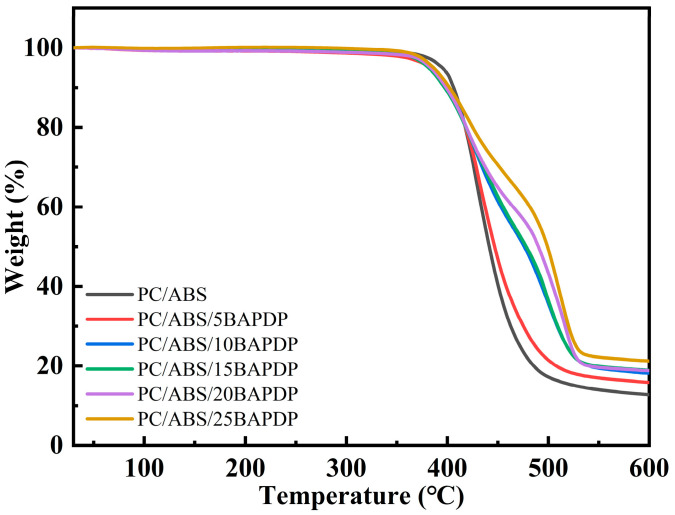
TGA curves of PC/ABS and flame-retardant PC/ABS composites.

**Figure 6:**
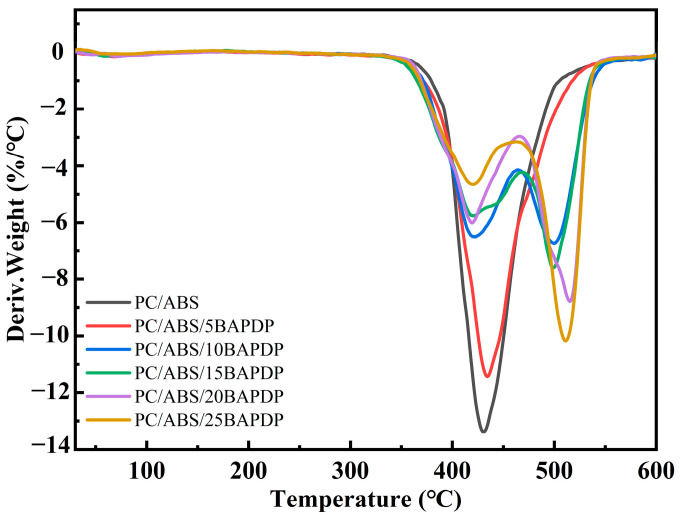
DTG curves of PC/ABS and flame-retardant PC/ABS composites.

The TGA curves of the PC/ABS composites (Figure 5) show that the addition of the flame retardant BAPDP decreased the temperature at which the PC/ABS alloy experienced a 5% weight loss (*T*_d5_) but increased the carbon residue content at 600 °C. With a BAPDP content of 15 wt%, *T*_d5_ was 381.4 °C, and the char yield at 600 °C was 18.9%, which was 47.7% higher than that of PC/ABS. Increasing the BAPDP content to 25 wt% further increased *T*_d5_ to 386.5 °C and the char yield at 600 °C to 21.2%, which was 65.6% higher than that of PC/ABS. This was attributed to the initial decomposition of BAPDP; however, this decomposition forms oxygen-containing acid, which forms phosphorus when heated. This phosphorus dehydrates PC/ABS, thereby increasing the carbon residue content at 600 °C. The results indicated that BAPDP exhibited strong charring ability and flame retardancy in the condensed phase. The max thermal mass loss rate (*T*_max_) of each of the composites with added BAPDP was higher than that of PC/ABS (Table 4). The *T*_max_ of PC/ABS/15BAPDP and the *T*_max_ of PC/ABS/25BAPDP were 68.5 and 81.4 °C higher than that of PC/ABS, respectively. The decomposition rate of the samples containing BAPDP was lower than that of PC/ABS (Figure 6), demonstrating that BAPDP improved the high-temperature thermal stability, thereby inhibiting its degradation. PC/ABS has a two-step degradation process—matrix thermal degradation (395–500 °C) and deep cracking, including chain cyclization and crosslinking (550–590 °C). However, after the addition of BAPDP, the primary degradation process of BAPDP occurs between the two degradation stages of PC/ABS, indicating that BAPDP may contribute throughout the thermal degradation process of the material. In Figure 6, the DTG shoulder peak of the PC/ABS/BAPDP samples decreased at 400–450 °C, and the char yield significantly increased at 600 °C (Table 4), indicating that BAPDP had a significant delay effect on the thermal decomposition of PC/ABS. Furthermore, the PC/ABS/BAPDP samples tended to deteriorate slightly at low temperatures, possibly because of minor PC/ABS chain scission in the early stages of TGA.

Cone calorimetry is one of the most effective methods to investigate the flammability of materials [35]. The relevant data, including the ignition time (TTI), peak heat release rate (PHRR), total heat release (THR), total smoke generation (TSP), total smoke release (TSR), and effective heat of combustion (EHC), are listed in Table 5.

**Figure 7:**
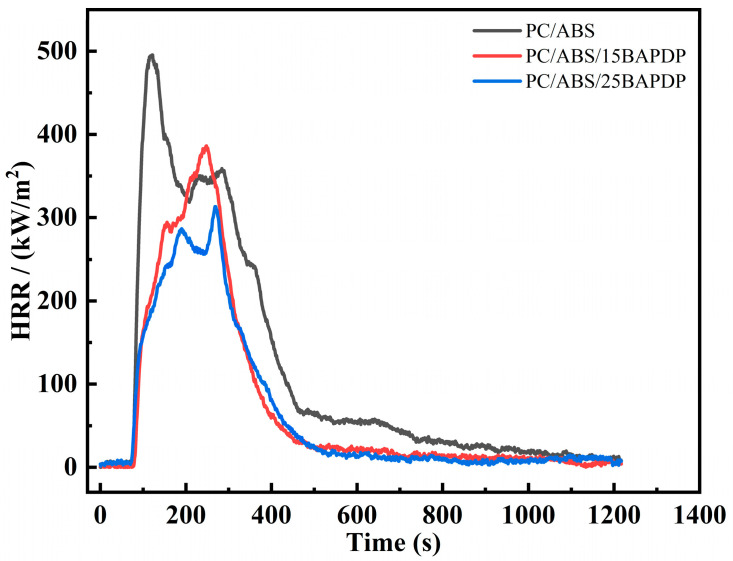
HRR curves of PC/ABS composites.

**Figure 8:**
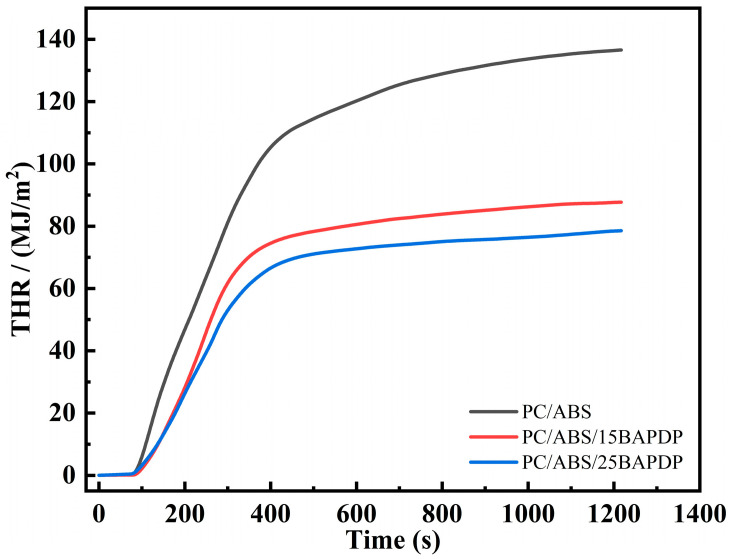
THR curves of PC/ABS composites.

The PHRR of PC/ABS/15BAPDP and PC/ABS/25BAPDP decreased by 22.1% and 36.8%, respectively, corresponding to that of PC/ABS (Table 5). This confirmed that BAPDP enhanced the inhibition of heat release in the flame-retardant system. The HRR and THR curves can be explained by the material properties (e.g., coke yield, EHC), sample effects (e.g., thickness, deformation), and physical properties that control the ignition behavior and combustion mechanism (e.g., carbon layer enhancement and fracture, reaction thermodynamics, release of different pyrolysis products) [36]. The introduction of BAPDP reduced both the heat release rate (HRR) and THR of the PC/ABS composites. The HRR and THR curves of the PC/ABS/15BAPDP and PC/ABS/25BAPDP curves were essentially below the PC/ABS curve (Figure 7 and Figure 8), and the addition of BAPDP significantly reduced the exothermic mass of the PC/ABS composite. The PHRR and THR of PC/ABS/15BAPDP were 386.2 kW/m^2^ and 87.9 MJ/m^2^, respectively, which were 22.1 and 35.7% lower, respectively, than those of PC/ABS (Table 5). Similarly, the PHRR (313.2 kW/m^2^) and THR (78.7 MJ/m^2^) of PC/ABS/25BAPDP were 36.8 and 42.4% lower, respectively, than those of PC/ABS. This confirmed that BAPDP suppressed heat release in the flame-retardant system. BAPDP may decompose into a series of phosphate esters at high temperatures, increasing the viscosity and strength of the participating carbon layer [37]. Therefore, the addition of BAPDP reduced the PHRR and THR of the composites.

**Figure 9:**
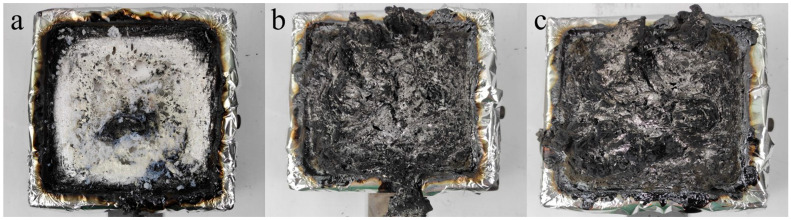
Photographs of (**a**) PC/ABS, (**b**) PC/ABS/15BAPDP, and (**c**) PC/ABS/25BAPDP after CCT.

To further investigate the flame-retardant effect of the PC/ABS/BAPDP composites, the surface and internal microstructures after CCT were characterized using optical photographs and SEM. In Figure 9, PC/ABS had the smallest amount of char formation among the samples, with more products dispersing into the air in the form of smoke during combustion. In comparison, PC/ABS/BAPDP had a higher degree of expansion, continuity, and integrity.

**Figure 10:**
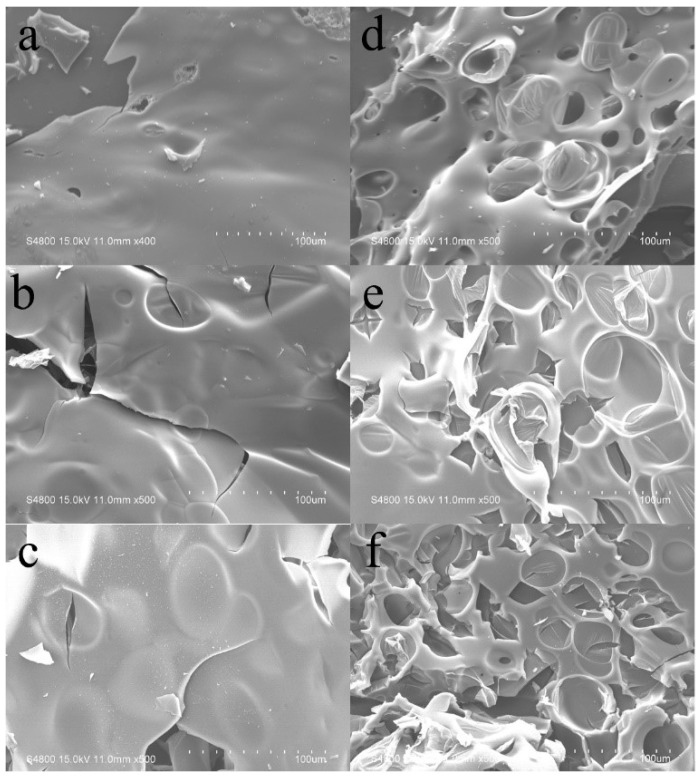
SEM images of the surface of char residues of PC/ABS composites after CCT: (**a**) PC/ABS, (**b**) PC/ABS/15BAPDP, and (**c**) PC/ABS/25BAPDP; SEM images of the internal char residues of PC/ABS composites after CCT: (**d**) PC/ABS, (**e**) PC/ABS/15BAPDP, and (**f**) PC/ABS/25BAPDP.

Figure 10 depicts the surface and internal micro-morphologies of the carbon residue in the PC/ABS systems after CCT. The char layer of the PC/ABS depicted several pores and exhibited low density owing to the extensive escape of various aromatic derivatives, CO_2_, and phenolic compounds during pyrolysis. The penetration of O_2_ through the loose and porous char layer exacerbated the burning process on the substrate. The addition of 15 and 25 wt% flame-retardant BAPDP enables the formation of a relatively hard carbon layer distributed on the combustion surface of the flame-retardant material (Figure 10b,c). BAPDP was heated to generate carbon from a phosphorus oxygen-containing acid catalytic polymer. This carbon was deposited on the surface of the material, protecting the internal matrix during combustion and inhibiting the flow of flammable gases and heat. Porous fluffy structures formed inside the carbon layers of PC/ABS/15BAPDP and PC/ABS/25BAPDP (Figure 10e,f), likely due to the flow of CO_2_ and other gases through the matrix upon heating BAPDP. The carbon layer inside PC/ABS/25BAPDP contained significantly more pores than that in PC/ABS/15BAPDP and significantly fewer irregular structures, resulting in a carbon residue with a more continuous and solid internal structure that better protected the internal matrix polymer. The carbon residue morphology was consistent with the TGA, indicating that BAPDP produces condensed flame retardancy. The flame-retardant BAPDP causes the formation of a dense continuous carbon layer that prevents the release of combustible gas and heat generation, thereby significantly reducing the total heat release.

### 3.3. Effects of BAPDP on Mechanical Properties of PC/ABS

The effects of BAPDP on the tensile, flexural, and notch impact strengths of the PC/ABS samples were measured to evaluate their mechanical properties. The tensile, flexural, and notch impact strengths of PC/ABS decreased with the addition of the flame retardant (Table 6). The tensile strength of PC/ABS with 15 wt% BAPDP decreased to 52.6 ± 1.3 MPa, which was 17.7% lower than that of PC/ABS (63.9 ± 1.9 MPa), while the flexural (83.1 ± 0.8 MPa) and notched impact (10.6 ± 0.8 kJ/m^2^) strengths were also lower than those of PC/ABS (93.4 ± 1.2 MPa and 25.5 ± 2.8 kJ/m^2^), respectively. BAPDP is a highly viscous liquid, which may inhibit its uniform dispersion through the PC/ABS matrix, reducing the notch impact strength. However, PC/ABS/BAPDP exhibited higher tensile, flexural, and notch impact strengths than the flame-retardant PC/ABS/RDP. This shows that PC/ABS/BAPDP has better mechanical properties than PC/ABS/RDP and can be used in a wider range of fields.

### 3.4. Effect of BAPDP on Thermal Deformation Temperature of PC/ABS

The thermal deformation temperatures (HDTs) of pure and flame-retardant PC/ABS are listed in Table 7. The HDT of the PC/ABS composites decreased with the increasing BAPDP content, because the BAPDP flame retardant served as a plasticizer and increased the polymer chain space. However, the HDT of PC/ABS/15BAPDP was 81.3 °C, which was 12.1 °C higher than that of PC/ABS/15RDP (69.2 °C). This shows that flame-retardant BAPDP had fewer adverse effects on the HDT of PC/ABS than the flame-retardant RDP. Therefore, BAPDP can expand the flame retardancy applications of PC/ABS in higher temperatures than RDP.

The comprehensive performance should be considered in the modification of PC/ABS with flame retardants; thus, the recommended dosage of BAPDP in PC/ABS is 20–25 wt%. This produces PC/ABS/BAPDP composites with a LOI > 25.4%, which achieves the UL-94 V-0 grade while maintaining relatively good mechanical properties, with a HDT > 69.7 °C. Compared with PC/ABS/RDP, PC/ABS/BAPDP has a better flame retardancy, mechanical properties, and heat resistance. Thus, BAPDP can expand the flame retardancy applications of PC/ABS.

### 3.5. Flame-Retardant Mode of Action for BAPDP in PC/ABS Blends

**Figure 11:**
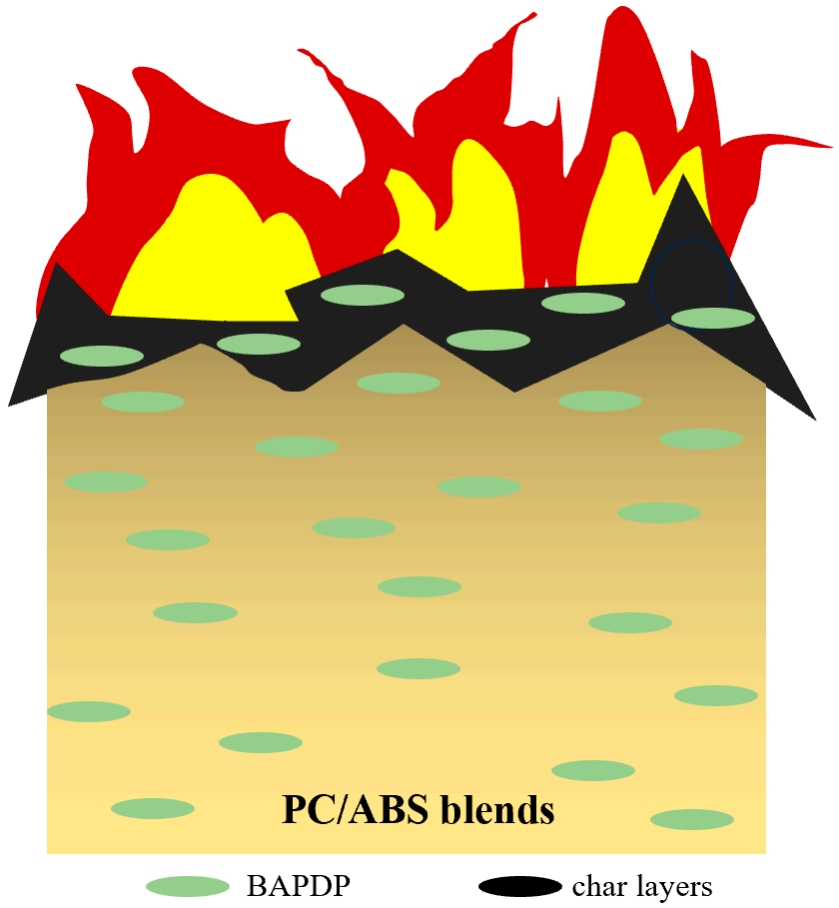
Flame-retardant mode of action for PC/ABS blends.

The afore-described experimental results show consistent accuracy; Figure 11 presents a flame-retardant mode of action for BAPDP in PC/ABS blends. In general, thermal degradation occurs in the first stage when the external heat source is continuously heated. Oxygen at the surface of the degrading polymer significantly promotes the thermos-oxidative process. The degrading polymer then continuously produces volatile combustibles, which undergo ignition when they reach a certain concentration and temperature. A portion of the heat released by combustion further feeds the degrading polymer. In this study, by melt blending, BAPDP was dispersed homogenously into PC/ABS blends. During combustion, the addition of BAPDP helps to form a continuous carbon layer that acts as a protective layer for the underlying material. This is because the phosphorous groups of BAPDP, which positively participate in the reactions of PC/ABS pyrolysis into char, promote the formation of a dense, stable carbon layer on the PC/ABS surface that further insulates heat and oxygen, thereby achieving a flame-retardant effect.

## 4. Conclusions

In this study, a novel flame-retardant BAPDP was synthesized via the sodium-phenolate-catalyzed transesterification of triphenyl phosphate and bisphenol AP. The molecular structure and thermal stability of BAPDP were characterized by FT-IR, ^1^H-NMR, TGA, and element analysis. The molecular structure of BAPDP contains numerous benzene rings and a high phosphorus content (8.2%), resulting in superior thermal stability and flame retardancy. The flame-retardant PC/ABS blends with BAPDP show strong flame retardancy, heat resistance, and mechanical properties. By adding 20 wt% BAPDP, the PC/ABS blends exhibited LOI values of 25.4%, a V-0 vertical burning grade, and a thermal deformation temperature of 72.6 °C. The CCT showed that BAPDP decreased the PHRR and THR of the material. Additionally, the flame retardancy, mechanical properties, and thermal deformation temperature of PC/ABS/BAPDP were superior to those of PC/ABS/RDP. This indicates that BAPDP can better balance the flame retardancy and heat resistance of PC/ABS than RDP. This study provides a basis for the further development of flame-retardant PC/ABS.

## Figures and Tables

**Table 1:** The compositions of the blended PC/ABS/BAPDP and PC/ABS/RDP composites.

Sample	PC/ABS (wt%)	BAPDP (wt%)	RDP (wt%)	PTFE (wt%)
PC/ABS	100	0	0	0
PC/ABS/5BAPDP	95	5	0	0.4
PC/ABS/10BAPDP	90	10	0	0.4
PC/ABS/15BAPDP	85	15	0	0.4
PC/ABS/20BAPDP	80	20	0	0.4
PC/ABS/25BAPDP	75	25	0	0.4
PC/ABS/15RDP	85	0	15	0.4
PC/ABS/20RDP	80	0	20	0.4
PC/ABS/25RDP	75	0	25	0.4

**Table 2:** BAPDP elemental analysis results.

Element	Experimental Content (%)	Theoretical Content (%)
C	68.2	70.0
H	5.0	4.8
P	8.1	8.2

**Table 3:** Effect of BAPDP and RDP contents on the LOI and UL-94 of flame-retardant PC/ABS.

Sample	BAPDP (wt%)	RDP (wt%)	LOI (%)	UL-94	*t*_1_/s	*t*_2_/s	Dripping
PC/ABS	0	0	21.1 ± 0.1	NR	>60	>60	Yes
PC/ABS/5BAPDP	5	0	22.3 ± 0.1	NR	>60	>60	Yes
PC/ABS/10BAPDP	10	0	23.1 ± 0.2	V-2	18.3	27.1	Yes
PC/ABS/15BAPDP	15	0	24.7 ± 0.1	V-1	15.6	19.1	No
PC/ABS/20BAPDP	20	0	25.4 ± 0.1	V-0	9.8	8.3	No
PC/ABS/25BAPDP	25	0	26.3 ± 0.1	V-0	7.8	6.6	No
PC/ABS/15RDP	0	15	24.1 ± 0.1	V-2	19.2	28.7	Yes
PC/ABS/20RDP	0	20	24.8 ± 0.1	V-1	16.3	19.8	No
PC/ABS/25RDP	0	25	25.7 ± 0.1	V-0	9.6	7.9	No

**Table 4:** TGA of PC/ABS and flame-retardant PC/ABS.

Sample	*T*_d5_ (°C)	*T*_max_ (°C)	Char Yield at 600 °C (%)
PC/ABS	395.2	426.8	12.8
PC/ABS/5BAPDP	383.5	431.1	15.8
PC/ABS/10BAPDP	385.8	499.5	18.2
PC/ABS/15BAPDP	381.4	495.3	18.9
PC/ABS/20BAPDP	383.5	516.5	18.8
PC/ABS/25BAPDP	386.5	508.2	21.2

**Table 5:** The CCT results of the samples.

Specimen	TTI (s)	PHRR (kW/m^2^)	THR (MJ/m^2^)	TSP (m^2^)	TSR (m^2^/m^2^)	Mean EHC (MJ/kg)
PC/ABS	75	495.5	136.6	19.7	2209.8	32.1
PC/ABS/15BAPDP	80	386.2	87.9	24.0	2691.0	24.9
PC/ABS/25BAPDP	72	313.2	78.7	28.0	2824.5	21.6

**Table 6:** Mechanical properties of PC/ABS and flame-retardant PC/ABS.

Sample	Tensile Strength, MPa	Flexural Strength, MPa	Izod Impact Strength, kJ/m^2^
PC/ABS	63.9 ± 1.9	93.4 ± 1.2	25.5 ± 2.8
PC/ABS/5BAPDP	58.3 ± 1.4	86.7 ± 0.9	15.2 ± 1.7
PC/ABS/10BAPDP	57.6 ± 1.1	85.2 ± 0.9	13.1 ± 1.8
PC/ABS/15BAPDP	52.6 ± 1.3	83.1 ± 0.8	10.6 ± 0.8
PC/ABS/20BAPDP	47.8 ± 1.2	78.7 ± 0.7	8.5 ± 0.7
PC/ABS/25BAPDP	40.7 ± 2.2	75.9 ± 0.9	7.3 ± 0.8
PC/ABS/15RDP	34.2 ± 0.4	80.1 ± 1.1	6.4 ± 0.3
PC/ABS/20RDP	28.2 ± 1.2	73.2 ± 0.8	4.9 ± 0.4
PC/ABS/25RDP	22.7 ± 1.3	70.9 ± 0.9	3.6 ± 0.3

**Table 7:** Thermal deformation temperature of PC/ABS and flame-retardant PC/ABS.

Sample	HDT (°C)
PC/ABS	111.6 ± 2.0
PC/ABS/5BAPDP	87.0 ± 0.6
PC/ABS/10BAPDP	83.8 ± 0.3
PC/ABS/15BAPDP	81.3 ± 0.7
PC/ABS/20BAPDP	72.6 ± 0.7
PC/ABS/25BAPDP	69.7 ± 1.3
PC/ABS/15RDP	69.2 ± 0.6
PC/ABS/20RDP	60.7 ± 0.6
PC/ABS/25RDP	57.2 ± 0.3

## Data Availability

The original contributions presented in the study are included in the article, further inquiries can be directed to the corresponding author.
